# Removal of polychlorinated naphthalenes by desulfurization and emissions of polychlorinated naphthalenes from sintering plant

**DOI:** 10.1038/srep26444

**Published:** 2016-05-20

**Authors:** Mengjing Wang, Wenbin Liu, Meifang Hou, Qianqian Li, Ying Han, Guorui Liu, Haifeng Li, Xiao Liao, Xuebin Chen, Minghui Zheng

**Affiliations:** 1 State Key Laboratory of Environmental Chemistry and Ecotoxicology, Research Center for Eco-Environmental Sciences, Chinese Academy of Sciences, Beijing 100085, China; 2 University of the Chinese Academy of Sciences, No. 19A Yuquan Road, Beijing 100049, China; 3 School of Ecological Technology and Engineering, Shanghai Institute of Technology, Shanghai 201418, China

## Abstract

The sintering flue gas samples were collected at the inlets and outlets of the desulfurization systems to evaluate the influence of the systems on PCNs emission concentrations, profiles, and emission factors. The PCNs concentrations at the inlets and outlets were 27888–153672 pg m^−3^ and 11988–42245 pg m^−3^,respectively. Desulfurization systems showed excellent removal for PCNs, and the removal efficiencies of PCNs increase with increasing chlorination level. Lower chlorinated homologs are more sensitive to the desulfurization process than higher ones. High levels of PCNs were also detected in the gypsum (11600–29720 pg g^−1^) and fly ash samples (4946–64172 pg g^−1^). The annual total emissions of PCNs released to flue gas and gypsum from the sintering plants were about 394 kg, 48.5% of which was in gypsum. The surface area of the fly ash samples increased significantly from the first to the fourth stage of the series-connected electrostatic precipitator, accompanying obvious rising of concentration of PCNs in the fly ash samples.

Polychlorinated naphthalenes (PCNs) are structurally (see Supplementary Fig. S1) and toxicologically similar to polychlorinated dibenzo-p-dioxins/dibenzofurans (PCDD/Fs), which pose potential risks to the global environment and human health^1^,^2^,^3^,^4^,^5^. PCNs could induce aryl hydrocarbon receptor-mediated responses, and thus display toxic mechanisms similar to those of dioxins^6^,^7^,^8^. The toxic equivalency factors (TEFs) of several PCN congeners relative to 2,3,7,8-tetrachlorodibenzo-*p*-dioxin (2,3,7,8-TCDD) have been studied^9^. Noma *et al.* summarized the TEFs of PCN congeners in 2004^10^, and Falandysz *et al.* recently evaluated and updated the relative potency factors of PCN congeners relative to 2,3,7,8-TCDD^11^. The persistent organic pollutant (POP) review committee (POPRC) has reviewed PCNs according to the screening criteria in Annex D of the Stockholm Convention on POPs. The POPRC concluded that di- to octachlorinated homologs of PCNs meet the screening criteria of POPs. Therefore, PCNs are listed as POPs and are targeted for elimination of their unintentional emissions and their manufacture and use in technical formulations are banned.

The manufacture and use of technical PCN formulations has almost ceased globally. Consequently, unintentional emission of PCNs from industrial thermal processes will become a more important PCN source than their manufacture. The levels of PCNs emitted from a number of industrial thermal processes have been reported^12^, including from waste incinerators, iron- and steel-making plants, hot dip galvanizing plants, and nonferrous smelting^3^,^9^,^13^,^14^,^15^,^16^,^17^,^18^. Identification of potential sources of unintentional PCNs is an essential primary step for evaluating source priority and implementing emission controls. Thus, it is important to identify potential industrial sources, particularly from industries with intensive activity in developing countries, and to estimate and characterize their PCN emissions.

China is the largest producer of sintering ore (964 million tons in 2012), accounting for about 40% of global sintering production. There are more than 500 sintering plants currently operating in China. Studies of PCN emissions from Chinese sintering plants would facilitate the evaluation of PCN release from iron ore sintering processes worldwide. Few investigations on PCN emissions from the sintering industry have been carried. A preliminary investigation of the iron ore sintering process was conducted by Liu *et al.*^19^. They found the emission concentrations in sintering flue gas were between 3 and 983 ng m^−3^ (0.4–23.3 pg TEQ m^−3^), and indicates that sintering process might be an important source of PCNs^19^.

The emission of SO_2_ from sintering processes in China has reached more than 140 million tons, accounting for 7.3% of total industrial emissions. Consequently, desulfurization systems have been introduced for air pollution control by removing SO_2_ from flue gases produced in sintering plants. Currently, wet and semi-dry desulfurization systems dominate in the desulfurization systems in sintering plants in China. Desulfurization systems can be used for synergistic control of various pollutants, including dust, SO_2_, and NO_x_^20^. However, there are few reports of systematic studies of simultaneous removal of PCNs from sintering flue gas using desulfurization systems.

The aim of the present study was to conduct a systematic study of PCN removal using desulfurization. Flue gas samples were collect from four representative sintering plants in China that operated on different industrial scales and used different desulfurization processes. The flue gas samples were collected at the desulfurization systems inlets and outlets to evaluate the influence of the systems on PCNs emission concentrations, profiles, and emission factors desulfurization. In addition, fly ash and gypsum samples were collected to improve the understanding of PCNs emissions from the sintering industry.

## Results

### Concentrations of PCNs in the flue gas, gypsum and fly ash samples

In China, small (<90 m^2^) sintering plants are mostly equipped with wet desulfurization systems, while medium (90–180 m^2^) and large (>180 m^2^) plants tend to be equipped with semi-dry desulfurization systems. As a representative sample, four sintering plants DH, TS, SK, and ST with areas of 90, 180, 360, and 500 m^2^, respectively, were selected for use in this study. The smallest plant (DH) was equipped with a wet desulfurization system, while the larger TS, SK, and ST plants were equipped with semi-dry desulfurization systems. The concentrations of di- to octachlorinated homologs of the PCNs (PCNs), dioxin-like Σ_2–8_PCNs (dl-PCNs) and Σ_2–8_PCNs TEQs (TEQs) were summed for each of the sintering plants (Table 1).

The ranges for the concentrations of PCNs and dl-PCNs in the flue gas at the desulfurization systems inlets were 27888–153672 pg m^−3^ (average 63990 ± 37081 pg m^−3^) and 4128–45030 pg m^−3^ (average 23858 ± 11029 pg m^−3^), respectively. For the outlets, the ranges for the concentrations of PCNs and dl-PCNs decreased to 11988–42245 pg m^−3^ (average 25324 ± 9968 pg m^−3^) and 25–16237 pg m^−3^ (average 7066 ± 4788 pg m^−3^). The ranges for the concentrations of PCNs and dl-PCNs in the gypsum samples were 11600–29720 pg g^−1^ (average 18590 ± 8310 pg g^−1^) and 3060–11100 pg g^−1^ (average 6800 ± 3550 pg g^−1^), respectively. The ranges for the PCNs and dl-PCNs concentrations in fly ash samples from sintering plants were 4946–64172 pg g^−1^ (average 19464 ± 17521 pg m^−3^) and 2455–18658 pg g^−1^ (average 5752 ± 4908 pg m^−3^), respectively.

The TEFs of several PCN congeners from Noma *et al.* were used for the calculation of TEQs^10^. The range for the TEQs at the inlets was 1.25–20.55 pg TEQ m^−3^ (average 7.01 ± 6.79 pg TEQ m^−3^). The range for the TEQs at the outlets was 0.14–6.39 pg TEQ m^−3^ (average 1.75 ± 1.80 pg TEQ m^−3^). The range for the TEQs in the gypsum samples was 1.1–2.8 pg TEQ g^−1^ (average 1.6 ± 0.83 pg TEQ m^−3^), and that in the fly ash samples was 0.7–6.3 pg TEQ g^−1^ (average 2.78 ± 1.83 pg TEQ m^−3^).

## Discussion

**Figure 1:**
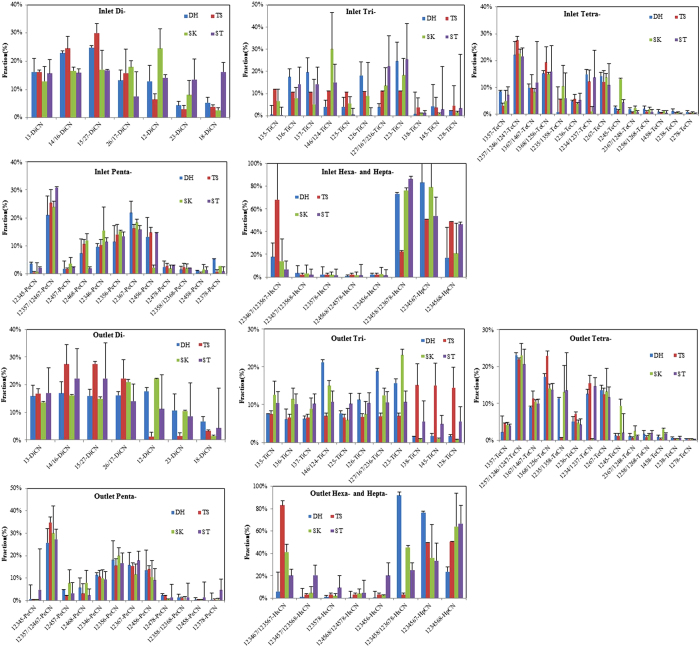
Congener profiles of PCNs in the flue gas samples at the inlets and outlets.

To compare the abundances of different PCN congeners for a homolog in the flue gas from the sintering plants, the concentrations of PCN congeners were calculated as proportions of the corresponding homolog concentration. The results for the PCN congeners in flue gas at the inlets are presented in Fig. 1.

Overall, the PCN congeners showed similar profiles at the inlets of all the sintering plants. The similarity in the relative abundances of the PCNs congeners at the inlets suggests that a similar mechanistic pathway results in the formation of PCNs at all the sintering plants. Furthermore, this suggests that the emission of PCNs is not related to the operating conditions or raw materials. The profiles for the PCN congeners emitted from the sintering processes were similar to the profiles for those emitted from combustion and metallurgical sources. However, they were different from the profiles in technical PCN mixtures.

Comparison of the relative abundances of the congeners for the DiCN to HpCN homologs at the inlets showed that chlorine substitutions at the α-positions of the naphthalene ring were unfavorable compared with substitution at the β-positions. The relative abundances of the congeners for the TriCN to HpCN homologs produced during sintering were generally in good agreement with the density functional theory which was investigated by Zhai *et al.*^21^.

For the DiCN and TriCN homologs, 1,5/2,7-DiCN (16.8–30.0%) and 1,2,3-TriCN (11.2–25.3%) were the most abundant. The relative abundances of the 1,8-DiCN and 1,2,8-TriCN congeners in sintering flue gas were very low, and these congeners were also had very low abundances in samples from waste incineration and iron industries^12^,^22^. For TeCN, the 1,2,5,7/1,2,4,6/1,2,4,7-chlorinated congeners were more abundant (21.6–27.6%) than other congeners in flue gas. For sintering, with increasing chlorination of the PCNs, the relative abundances of congeners with more chlorines at the β-positions were much higher than those of congeners with more chlorines at the α-positions. For example, the relative abundances of the 1,2,3,5,7/1,2,4,6,7-, 1,2,3,6,7-chlorinated congeners (three chlorines in the β-position) were much higher than those of the 1,2,4,5,7- and 1,2,4,6,8-chlorinated congeners (three chlorines in the α-position). Similar trends for the relative abundances of the congeners were observed for HxCN and HpCN homologs. The 1,2,3,4,6,7-/1,2,3,5,6,7-chlorinated congeners with up to four β-position were the most abundant for HxCN. For HpCN, the relative abundance of the 1,2,3,4,5,6,7-chlorinated congener with four β-position chlorines was much higher than that of the 1,2,3,4,5,6,8-chlorinated congener with four α-position chlorines.

The effect of PCN congener profiles by desulfurization were presented in Fig. 1. The 1,4/1,6-DiCN, 1,2,3-TiCN, 1,2,5,7/1,2,4,6/1,2,4,7-TeCN, 1,2,3,5,7/1,2,4,6,7-PeCN, 1,2,3,4,6,7/1,2,3,5,6,7-HxCN, and 1,2,3,4,5,6,8-HpCN congeners were the most abundant for the DiCN, TriCN, TeCN, PeCN, HxCN, and HpCN homologs, respectively. The profiles for the relative abundances of the PCN congeners at the outlets were basically consistent with the profiles at the inlets and the desulfurization systems had little influence on the congeners, except for HxCN and HpCN. From the inlets to the outlets of the semi-dry desulfurization systems, the relative abundances of 1,2,3,4,6,7/1,2,3,5,6,7-HxCN increased by 8.4% and those of 1,2,3,4,5,8/1,2,3,6,7,8-HxCN decreased by 36.9%. For the wet desulfurization systems, the relative abundances of 1,2,3,4,6,7/1,2,3,5,6,7-HxCN decreased by 11.8% and those of 1,2,3,4,5,8/1,2,3,6,7,8-HxCN increased by 18.7% from the inlets to the outlets. For HpCN, the relative abundances of 1,2,3,4,5,6,7-HpCN decreased by 21.5% and 6.6% from the inlets to the outlets of the semi-dry and wet desulfurization systems, respectively. For 1,2,3,4,5,6,8-HpCN, the relative abundances increased by 21.5% and 6.6% from the inlets to the outlets of the semi-dry and wet desulfurization systems, respectively. The differences observed in the relative abundances of 1,2,3,4,6,7/1,2,3,5,6,7-HxCN, 1,2,3,4,5,8/1,2,3,6,7,8-HxCN, and 1,2,3,4,5,6,7-HpCN and 1,2,3,4,5,6,8-HpCN suggests that these congeners are more sensitive than the other targeted species to semi-dry and wet desulfurization. Liu *et al.* found that 1,2,3,4,5,6,7-HpCN was much more abundant than the other HpCN congeners in emissions from thermal-related sources^3^, and the character is considered to be the main indicator of PCN emissions from combustion and metallurgical plants. However, it is noteworthy that the 1,2,3,4,5,6,7-HpCN concentration was lower than the 1,2,3,4,5,6,8-HpCN concentration in the flue gas at the semi-dry desulfurization system outlets, which differs from the conclusion of Liu *et al.*^3^.

The adsorption of PCNs congener profiles by gypsum (see Supplementary Fig. S2) from the wet and semi-dry desulfurization systems were similar. Lower chlorinated homologs showed relatively different distribution patterns to higher chlorinated homologs. The relative abundances of 1,3-DiCN in gypsum samples from wet desulfurization were higher than those in gypsum from semi-dry desulfurization, and the relative abundances of 1,4/1,6-, 1,5/2,7-, 2,6/1,7-DiCN in wet desulfurization gypsum samples were lower than those in semi-dry desulfurization gypsum samples. For TriCN, the relative abundances of the 1,4,5- and 1,2,8-chlorinated congeners in gypsum from the wet desulfurization systems were lower than those in gypsum from the semi-dry desulfurization systems. However, for other TriCN congeners in the gypsum samples from the wet desulfurization system, the relative abundances were higher than those in the semi-dry desulfurization gypsum samples. The relative abundance of 1,2,3,4,5,6,7-HpCN was higher than of 1,2,3,4,5,6,8-HpCN in gypsum from the wet desulfurization systems, while the relative abundance of 1,2,3,4,5,6,7-HpCN was lower than that of 1,2,3,4,5,6,8-HpCN in gypsum from semi-dry desulfurization.

The relative toxic contributions of the PCN congeners in flue gas, gypsum, and fly ash samples are shown in see Supplementary Fig. S4. The most toxic congener at the inlets and outlets of the DH, SK, and ST plants was 1,2,3,4,5,8/1,2,3,6,7,8-HxCN because it had a relatively high REF compared with the other PCN congeners. For the TS plant, the most toxic congener was 1,2,3,4,6,7/1,2,3,5,6,7-HxCN at the inlets and outlets because it was present at much higher concentrations than the other PCN congeners. From the inlet to the outlet, the TEQ of 1,2,3,4,6,7/1,2,3,5,6,7-HxCN decreased with wet desulfurization, and increased with semi-dry desulfurization. For 1,2,3,4,5,8/1,2,3,6,7,8-HxCN, the TEQ increased with wet desulfurization and decreased with semi-dry desulfurization. The toxicity contributions of the congeners in the gypsum samples from those four plants were similar, and 1,2,3,4,5,7/1,2,3,5,6,8-HxCN was the largest contributor to the TEQs.

**Figure 2:**
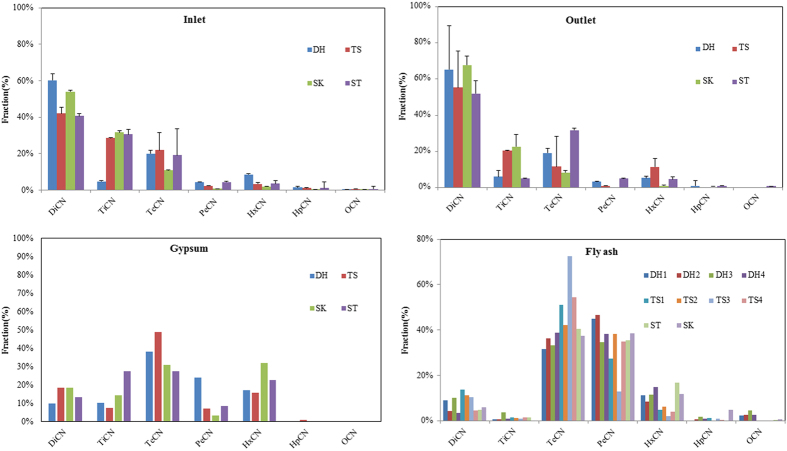
Homolog profiles of PCNs in flue gas, gypsum and fly ash samples from sintering plants.

Knowledge of the distribution of PCN homologs could be used to understand the formation and removal mechanisms of PCNs. In this study, the homolog concentrations were normalized to the total PCN. PCN homologs distribution in the flue gas samples from sintering processes are shown in Fig. 2.

Generally, successive reductions of homologues (through di- to octa- homologs) were observed as the number of chlorine substituents increased, which indicates that chlorination might be an important pathway for PCN formation^12^. The homolog patterns at the inlets were dominated by the DiCN homologs (40.6–60.3% of the PCNs), followed by the TriCN and TetraCN homologs (4.8–31.7% and 10.7–22.0%, respectively). The proportions of DiCN, HxCN, PeCN, and HpCN homologs at the inlets in the small DH plant were higher than those from the three larger TS, SK, and ST plants. However, the proportion of TriCN at the inlet in the DH plant was much lower than the proportions at the inlets in the three bigger sintering plants. The differences observed in the proportions of these six homologs are more sensitive than the other targeted species to the operating conditions and the raw materials.

After the effect of PCN homologs distributions by desulfurization, the homolog patterns at the outlets were dominated by DiCN homologs (Fig. 2), with the proportion of this homolog (range 52.0–65.3%) increasing compared with the proportion at the inlet for all four sintering plants. The proportion of higher chlorinated homologs (HpCN and OcCN) decreased at the outlets compared with the inlets, which could be attributed to the higher removal efficiency of the desulfurization system for higher chlorinated homologs than for lower chlorinated PCN homologs. Different desulfurization systems have different removal efficiencies for lower chlorinated PCNs. In the present study, the proportion of DiCN at the outlet increased by 5.0% compared with that at the inlet for sintering plants with wet desulfurization systems outlets and increased 12.7% at the semi-dry desulfurization systems. The proportion of TriCN at the outlet increased by 1.2% compared with that at the inlet for sintering plants with wet desulfurization systems, and by 14.3% for those with semi-dry desulfurization systems. These results suggest that lower chlorinated homologs are more sensitive to the desulfurization process than higher chlorinated homologs.

Adsorption of PCNs homologs distribution in the gypsum samples (Fig. 2) were dominated by TeCN (27.7–49.0%), followed by the HxCN (16.0–32.2%). The proportions of TeCN and HxCN were higher than those of DiCN (average 15.1%) and TriCN (average 15.1%), which shows that more TeCN and HxCN were removed from the flue gas and transferred to gypsum than DiCN and TriCN. The proportions of PeCN, HpCN and OcCN homologs in the gypsum samples were much lower than those of the other homologs because they were present at much lower concentrations in the sintering flue gas.

The degree of chlorination (Table 1) is calculated as the sum of the homolog proportion multiplied by the number of chlorine substituents in the homolog. It is an important parameter that can be used to evaluate the distribution of different chlorinated homologs^22^. The range for the degree of chlorination for the PCNs at the inlets was 2.47–3.53, and this decreased to 1.99–3.49 at the outlets. This is because the lower chlorinated homologs contributed relatively high proportions of the total PCNs concentrations in the desulfurization systems. The degree of chlorination in the sintering plants was comparable to that observed in the coking industry and in waste incinerators^15^,^18^. The range for the degree of chlorination in the gypsum samples was 3.99–4.29.

The concentrations of the PCNs varied considerably among the different sintering plants (Table 1). The concentration of PCNs emitted from the DH plant was higher than those at the other plants. This could be affected be two factors. First, the DH sintering plant was smaller than the other plants and had outdated equipment, which is widely recognized to contribute to PCN emissions^23^. Second, the raw materials would contribute to the formation and release of POPs^24^. Xhrouet *et al.* reported that dust accounted for 10% of the raw material collected in an electrostatic precipitator, which lead to a 1000-fold increase in the amount of PCDD/Fs formed^25^. In the present study, the proportion of recycled material in the raw materials used at the DH sintering plant (>20%) was much higher than that at most of the other plants, which might account for the higher concentration of PCNs observed for this plant.

Comparing PCN emissions from sintering plants with other those from industrial thermal processes could aid understanding of the environmental PCN burdens and be used to prioritize PCN sources. Most available PCN data are for waste incineration (97600–874000 pg m^−3^, 1.74–32.6 pg TEQ m^−3^), secondary metal smelting (41300–2245000 pg m^−3^, 0.07–0.30 pg TEQ m^−3^), converter steelmaking processes (1490–44300 pg m^−3^, 0.06–0.56 pg TEQ m^−3^), iron foundries (7339–104445 pg m^−3^, 0.49–1.90 pg TEQ m^−3^), and coking processes (1600–91800 ng m^−3^, 0.08–4.23 pg TEQ m^−3^)^14^,^15^,^26^,^27^. The PCN concentrations measured at the outlets in the present study were generally lower than those reported for waste incineration, secondary metal smelting, and iron foundries, and comparable to those from coking processes and converter steelmaking processes.

The concentrations of PCNs in gypsum have not been reported before. In the present study, the concentrations of PCNs in the gypsum samples were relatively high. Compared with other solid samples, the concentrations found in gypsum samples in the present study were much higher than those found soil around a municipal solid waste incinerator^28^, or in fly ash from the coking industry and converter steelmaking processes^27^,^29^.

**Figure 3:**
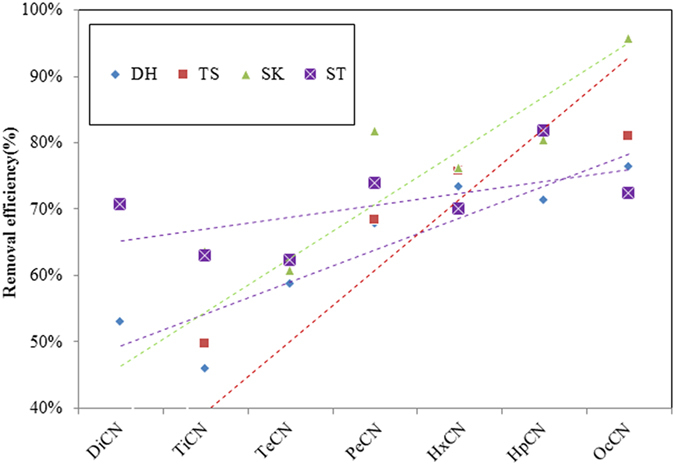
Desulfurization removal efficiencies for di- to octachlorinated homologs of the PCNs.

The desulfurization systems investigated in this study showed excellent removal efficiency of PCNs for the sintering process, and the PCNs concentrations at the inlets were higher than those at the outlets. The removed PCNs were adsorbed from the flue gas by gypsum. The ranges for the removal efficiencies of PCNs by the desulfurization systems were 54.5–69.1% for the PCN concentrations, 59.9–85.1% for the dl-PCN concentrations, and 66.5–85.0% for the TEQs. Figure 3 shows the removal efficiencies of the PCN homologs for the investigated sintering plants.

The removal efficiencies of the PCN homologs increased with increasing chlorination level. This indicates that desulfurization systems have higher removal efficiencies of higher chlorinated homologs than lower chlorinated homologs. Generally, lower chlorinated homologs have higher vapor pressures and higher gaseous fractions at a specific temperature than higher chlorinated homologs. Higher chlorinated homologs are more likely to condense on particulate matter than lower chlorinated homologs. Solid particles in sintering flue gas were intercepted by desulfurization systems, and this means the systems can effectively remove solid-phase PCNs, but are ineffective at removing gas-phase PCNs. The high chlorinated PCNs congeners have higher removal efficiency than low chlorinated.

The PCN, TEQ and dl-PCN concentrations in the fly ash samples clearly increased from the first to the fourth stages of the series-connected electrostatic precipitator in the DH and TS plants. The PCN concentrations in the fly ash samples were higher than those in the gypsum samples. The results were compared with those for PCNs in fly ash from iron foundries^12^, copper metallurgy^17^, the coking industry^29^, and converter steelmaking processes^27^. The concentrations and TEQs for the PCNs in fly ash from sintering in this study were generally lower than those reported for copper metallurgy, but higher than those from iron foundries, the coking industry, and converter steelmaking processes.

Comparing the characteristics of the fly ash samples from the different stages (see Supplementary Fig. S5), we found the particle size decreased and the Brunauer–Emmett–Teller surface area and total organic carbon content both increased from the first to the fourth stage of the series-connected electrostatic precipitator in each plant. This shows that the finer fly ash has a higher specific surface area, which favors adsorption of PCNs. The higher amount of organic carbon in the fly ash was related to higher levels of PCNs in the fly ash.

The PCN congener profiles (see Supplementary Fig. S3) and homolog patterns (Fig. 2) in the fly ash samples from the first to fourth stages in the series-connected electrostatic precipitators from those four plants were similar. The similarities in the relative abundances of the PCN congeners and homologs suggest that a similar mechanistic pathway results in the formation of PCNs in fly ash from all the different plants. It also suggests that the PCN emission patterns are not related to the use of a series-connected electrostatic precipitator. The PCN congener profiles and homolog patterns in the fly ash samples from the sintering plants were clearly different from those from coking process^29^. The congeners 1,3-DiCN, 1,4,5-TriCN, 1,3,6,8/1,2,5,6-TeCN, 1,2,3,5,6-PeCN, 1,2,3,4,6,7/1,2,3,5,6,7-HxCN, and 1234567-HpCN dominated the DiCN, TriCN, TeCN, PeCN, HxCN, and HpCN homologs, respectively. The homolog patterns were dominated by the TeCN and PeCN homologs. The toxicity contributions in fly ash samples were similar and comparable to those from gypsum samples. The dominate congener for the TEQs was 123457/123568-HxCN (see Supplementary Fig. S4). The degree of chlorination in fly ash samples from the fourth stage of the series-connected electrostatic precipitator in the DH (range 3.88–5.07) and TS (4.03–4.44) sintering plants and the degree of chlorination in fly ash samples from the SK and ST sintering plants were 3.93 and 4.81, respectively.

The emission factor of a contaminant is typically used for estimating total annual emissions and for establishing inventory. The desulfurization system effectively decreased the emission factor of PCNs and TEQs for the flue gas. The emission factor of PCNs decreased by 60.4% from 255 μg t^−1^ to 101 μg t^−1^, and the emission factor of TEQs decreased by 75.0% from 0.028 μg TEQ t^−1^ to 0.007 μg TEQ t^−1^. The emission factors of PCNs for fly ash and gypsum were 281 μg t^−1^ (0.044 μg TEQ t^−1^) and 198 μg t^−1^ (0.017 μg TEQ t^−1^). In China in 2012, the total output of sinter was 964 million tons, in which 48.6% of the sinter was equipped desulfurization systems. The total flue gas emissions of PCNs from the sintering process were estimated at about 173 kg (17.1 g TEQ). If all plants in China were not equipped with desulfurization systems, the emission of PCNs released to air would be up to 245 kg (27.0 g TEQ), and the emission of PCNs was reduced about 29.4% (on mass concentrations bases) and 36.6% (on TEQs bases) by desulfurization. The emission of PCN released to fly ash and gypsum was estimated at about 271 kg (42.6 g TEQ) and 191 kg (16.4 g TEQ). The fly ash produced in the sintering plants was recycled to recover residual metals, avoided the PCNs emission from fly ash. Thus, the total emissions of PCNs from sintering plants were estimated to be about 394 kg (33.5 g TEQ), indicating that PCN emitted to gypsum accounting for 48.5% and 49.0% on mass concentrations and TEQs basis, respectively. However, the PCNs in gypsum are not effectively eliminated, and they will probably reenter the environment and become a new emission source. Therefore, the risks of PCNs in gypsum should be prioritized and emission control sound management should be developed.

**Figure 4:**
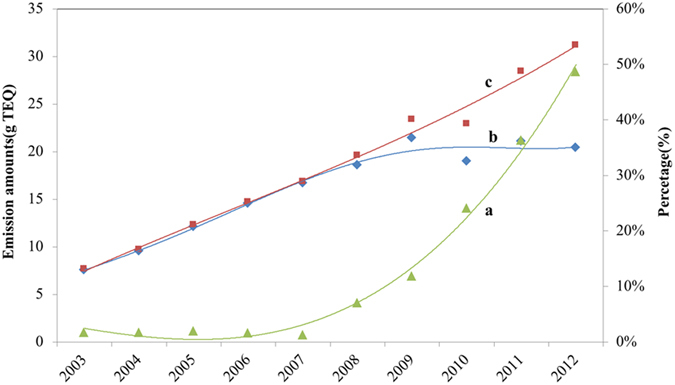
Estimation the emission of PCNs from sintering plants in 2003–2012. (**a**) The percentage of plants that had desulfurization systems in 2003–2012; (**b**) The actual emission of PCNs from sintering processes in 2003–2012; (**c**) The emission of PCNs that all sintering plants were not equipped with desulfurization systems in 2003–2012.

Combine the output of sintering (see Supplementary Fig. S6) and the percentage of plants that had desulfurization systems in 2003–2012 (Fig. 4a). The actual emission of PCNs from sintering processes in 2003–2012 (Fig. 4b) was preliminarily estimated to be about 138.2 g TEQ. In addition, all sintering plants which were not equipped with desulfurization systems in 2003–2012 were provided, the corresponding total emission of PCNs (Fig. 4c) was found to be 161.6 g TEQ. The gap between actual emissions and the emissions from plants which were not equipped with desulfurization systems get significantly large gradually increased as the percentage of plants with desulfurization systems increased. That is speculated that desulfurization may play key a role in controlling the PCNs emission.

## Methods

### Sample collection

**Figure 5:**
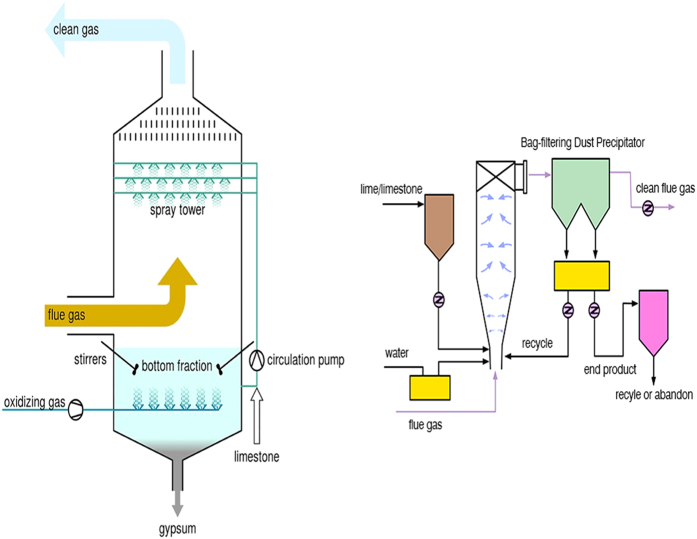
Schematic of (left) wet and (right) semi-dry desulfurization systems.

Schematics of wet and semi-dry desulfurization systems are shown in Fig. 5, and the operational information of investigated plants DH, TS, SK and ST are shown in Supplementary Table S1.

In a wet desulfurization system, the flue gas is in contact with a slurry of the sorbent in an aqueous medium. The nozzles and injection locations are designed to provide good contact between the flue gas and sorbent. Cleaned flue gas passes through a mist eliminator, which removes any slurry droplets, before emission into the atmosphere. A semi-dry desulfurization uses an aqueous sorbent slurry similar to that used in the wet system, but with a higher sorbent concentration. The waste product is collected with a standard particulate matter collection device such as a baghouse.

A series of flue gas, fly ash, and gypsum samples were collected from the sintering plants in June 2014. Six flue gas samples were collected from each sintering plant, with three flue gas samples collected at the desulfurization systems inlets and three at the outlets. Flue gas samples were collected using an automatic isokinetic sampling system (TCR TECORA, Italy), and the methods used for sample collection are described in detail in an earlier study^14^. Briefly, the sampling train included a heated probe, a filter box equipped with a quartz fiber filter, and a water-cooled XAD-2 adsorbent trap. The quartz fiber filter was used to collect particulate-bound pollutants, and the XAD-2 adsorbent resin was used for trapping vapor-phase contaminants. Four fly ash samples were collected at different stages in series-connected electrostatic precipitators from the DH and TS plants. One fly ash sample that was a mixture from all stages was collected from the electrostatic precipitators in the SK and ST plants. In addition, a gypsum sample was collected from the desulfurization system at each plant.

### Preparation and analysis of PCNs

The PCNs were analyzed using an isotope dilution high-resolution gas chromatography/high-resolution mass spectrometer as described previously^14^. The flue gas samples were spiked with 1 ng ^13^C_10_-labeled PCN internal standard mixture (ECN-5102, Cambridge Isotope Laboratories, Tewksbury, MA, USA) containing ^13^C_10_-1,2,3,4-TeCN, ^13^C_10_-1,3,5,7-TeCN, ^13^C_10_-1,2,3,5,7-PeCN, ^13^C_10_-1,2,3,5,6,7-HxCN, ^13^C_10_-1,2,3,4,5,6,7-HpCN, and ^13^C_10_-1,2,3,4,5,6,7,8-OCN). Fly ash and gypsum samples were digested in 1 mol L^−1^ HCl, rinsed with deionized water until a pH value close to 7.0 was reached, and then dried before Soxhlet extraction. Then, the samples were Soxhlet extracted for approximately 24 h. The extract was evaporated to dryness, and then cleaned by passing through a column packed with silica gel treated with 44% (mass fraction) sulfuric acid, a multilayer silica gel column, and a column packed with basic alumina. The volume of the final extract was 20 μL by rotary evaporation and under a gentle stream of nitrogen gas, and a ^13^C_10_-labeled PCN injection standard (ECN-5260; Cambridge Isotope Laboratories) containing ^13^C_10_-1,2,3,4,5,7/1,2,3,5,6,8-HxCN was added before instrumental analysis.

The target PCNs were analyzed by a gas chromatograph (GC) coupled with a DFS mass spectrometer (Thermo Fisher Scientific, Hudson, NH, USA) using an electron impact (EI) source. A DB-5 MS capillary column (60 m × 0.25 mm × 0.25 mm, Agilent Technologies, Santa Clara, CA, USA) was used to separate the PCN congeners. The electron energy was set to 45 eV. The source temperature was 270 °C. The high-resolution mass spectrometer was operated in selected ion monitoring mode with a resolution greater than 10,000. The GC oven program was as follows: held at 80 °C for 2 min, increased at a rate of 20 °C/min to 180 °C (held 1 min), increased at a rate of 2.5 °C/min to 280 °C, and increased at a rate of 10 °C/min to 290 °C (held 5 min). The injector and interface temperatures were set to 260 °Cand 290 °C, respectively. The carrier gas was helium at a flow rate of 1 mL/min. The sample size was 1 μL and samples were injected in split-less mode. Peaks corresponding to the individual PCN congeners were identified based on their retention times relative to those of internal standards and by their ion ratios.

### Quality assurance and quality control

Peaks were identified based on comparison of the retention times with those of available individual standards and ion ratios, and considering the elution order on the DB-5 column. Peaks were quantified if the GC retention times matched those of the standard compounds and the ratios of target/qualifier ion were within 15% of the theoretical values. The detection limits and quantification limits were defined as three and ten times the signal-to-noise ratio, respectively. The range for recoveries of ^13^C_10_-PCN internal standards relative to labeled injection standards was 62–99%. Blank tests were carried out every three samples.

## Additional Information

**How to cite this article**: Wang, M. *et al.* Removal of polychlorinated naphthalenes by desulfurization and emissions of polychlorinated naphthalenes from sintering plant. *Sci. Rep.* **6**, 26444; doi: 10.1038/srep26444 (2016).

## Supplementary Material

Supplementary Information

## Figures and Tables

**Table 1:** Concentrations and chlorination degree of PCNs in samples from the sintering plants.

			PCNs	dl-PCNs	dl-PCNs TEQ	Chlorination degree
Flue gas (pg m^−3^, pg TEQ m^−3^)	Inlet	DH	87972 ± 6570	32850 ± 1218	11.6 ± 6.74	3.02 ± 0.50
		TS	58180 ± 20024	30629 ± 1896	1.94 ± 0.60	3.02 ± 0.51
		SK	45087 ± 6193	10321 ± 6193	3.58 ± 2.03	2.67 ± 0.20
		ST	64720 ± 36832	21630 ± 2507	10.9 ± 9.65	3.06 ± 0.26
	Outlet	DH	40069 ± 2176	13168 ± 3069	3.89 ± 2.50	2.81 ± 0.14
		TS	17988 ± 6000	4572 ± 913	0.30 ± 0.05	2.94 ± 0.55
		SK	22910 ± 3577	3602 ± 3577	1.15 ± 1.01	2.45 ± 0.04
		ST	20327 ± 6314	6923 ± 4454	1.64 ± 0.15	3.11 ± 0.04
Gypsum (pg g^−1^, pg TEQg^−1^)	DH		11600	4900	2.8	42.9
	TS		12940	3060	1.2	39.9
	SK		29720	11100	1.1	41.7
	ST		20110	8150	1.1	39.9
Fly ash (pg g^−1^, pg TEQg^−1^)	DH	Stage1	4946	2455	1.51	5.07
		Stage2	8232	2559	1.59	4.80
		Stage3	9322	2660	2.00	3.88
		Stage4	16550	5657	4.92	4.81
	TS	Stage1	9504	2508	0.71	4.17
		Stage2	12414	4045	1.60	4.45
		Stage3	32040	5996	1.88	4.03
		Stage4	64172	18658	6.26	4.44
	SK (mixed)		20699	8071	2.65	3.93
	ST (mixed)		16764	4913	4.54	4.81
Calculated as ∑[(Homolog sum/∑PCNs)× No. of Cl].						

Concentrations of PCNs in samples from the sintering plants.
