# Supplementary material for: Removal of polychlorinated naphthalenes by desulfurization and emissions of polychlorinated naphthalenes from sintering plant
Source: Sci Rep. 2016 May 20;6:26444. doi: 10.1038/srep26444 (PMC4873742; doi:10.1038/srep26444)
Supplement: Supplementary Information [file srep26444-s1.pdf]

Supplementary Information

Removal of polychlorinated naphthalenes by desulfurization and emissions of polychlorinated naphthalenes from sintering plant

Mengjing Wang[1,2], Wenbin Liu[1]*, Meifang Hou[2], Qianqian Li[1], Ying Han[1], Guorui Liu[1], Haifeng Li[1], Xiao Liao[1], Xuebin Chen[1], Minghui Zheng[1]

[1] State Key Laboratory of Environmental Chemistry and Ecotoxicology, Research Center for Eco-Environmental Sciences, Chinese Academy of Sciences, Beijing 100085, China, University of the Chinese Academy of Sciences, No. 19A Yuquan Road, Beijing 100049, China

[2] School of Ecological Technology and Engineering, Shanghai Institute of Technology, Shanghai 201418, China

*Corresponding author: Dr. Wenbin Liu; Tel.: +86 10 62849356; Fax: +86 10 62849356; E-mail: liuwb@rcees.ac.cn

List of Contents:

Supplementary Table S1. Operational details for the sintering plants.

Supplementary Figure S1. PCN structure, with the α and β positions.

Supplementary Figure S2. Congener profiles of PCNs in the gypsum samples.

Supplementary Figure S3. Congener profiles of PCNs in the fly ash samples.

Supplementary Figure S4. TEQ congener patterns of PCNs in the flue gypsum and fly ash samples from sintering plants.

Supplementary Figure S5. Characteristics of fly ash samples from sintering plants. Size=particle size, BET=Brunauer–Emmett–Teller surface area, TOC=total organic carbon.

Supplementary Figure S6. The output of sintering in China (2003-2012).

Items		DH	TS	SK	ST
Sintering size (m^2)		90	180	360	500
Annual output of sintering ($\times 10^4$ ton)		80	220	400	695
Feed materials (%)	Iron ore	63.4	76	78	76
	Limestone	11.5	9	5	9.4
	Coke	5.4	9	4	4.9
	Recycled dust	20.1	6	13	9.7
Desulfurization process		Wet	Semidry	Semidry	Semidry
Average temperature of flue gas (°C)		57.4	133	149.2	182.7
Average flue gas flow (m^3 h^{-1})		420000	1100000	1810000	3400000
Output of gypsum(ton y^{-1})		9000	26000	40000	73000
Output of fly ash(ton y^{-1})		8700	20000	42000	59000

Supplementary Table S1. Operational details for the sintering plants.

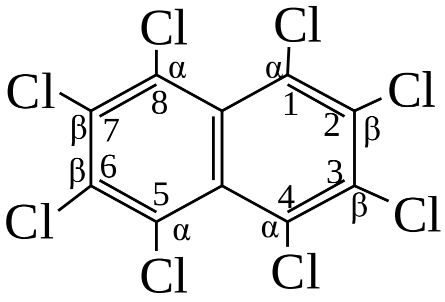

Supplementary Figure S1. PCN structure, with the α and β positions.

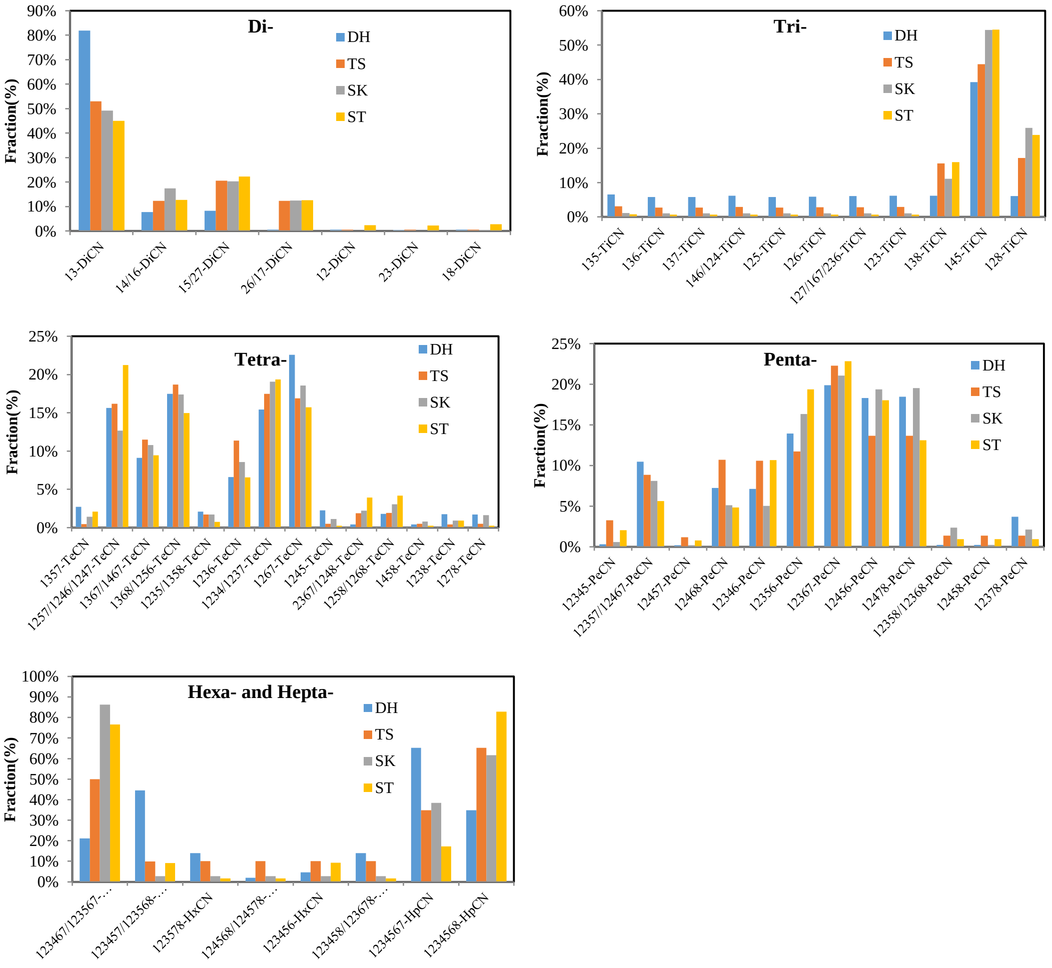

Supplementary Figure S2. Congener profiles of PCNs in the gypsum samples.

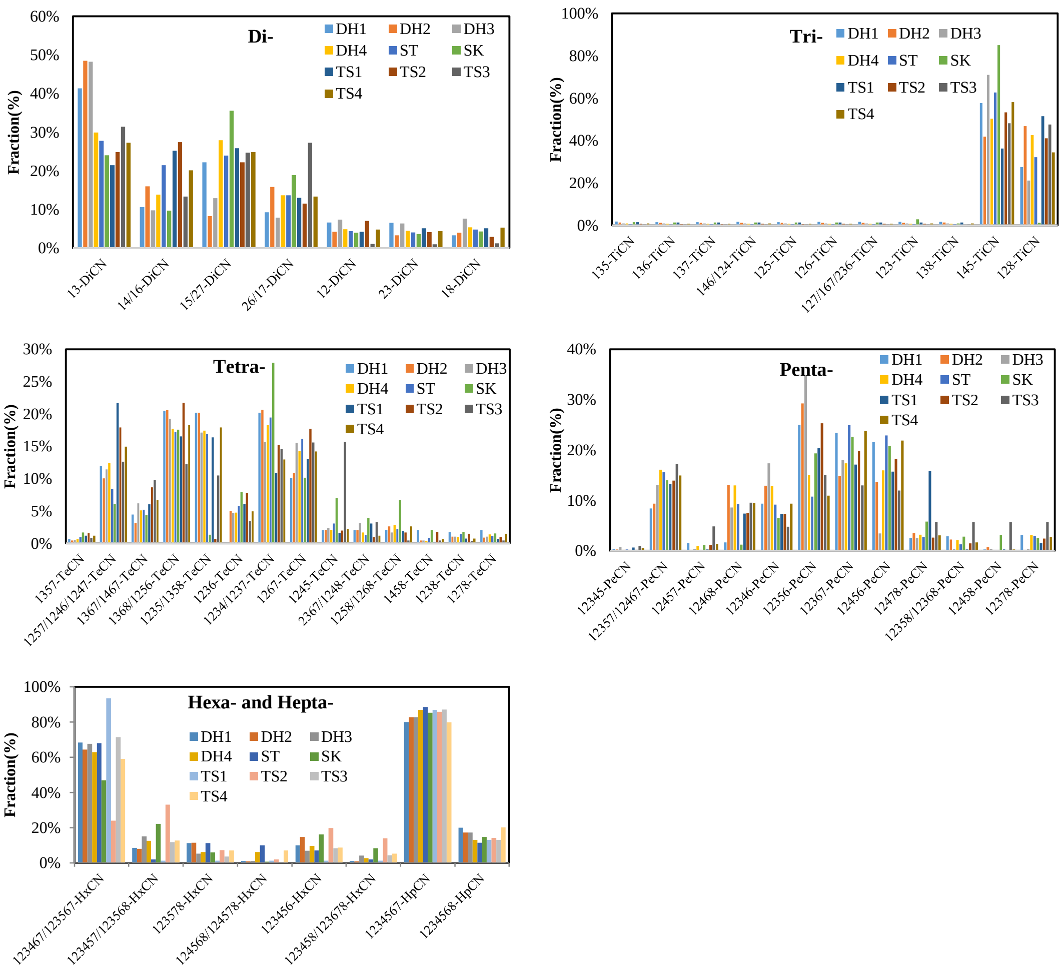

Supplementary Figure S3. Congener profiles of PCNs in the fly ash samples.

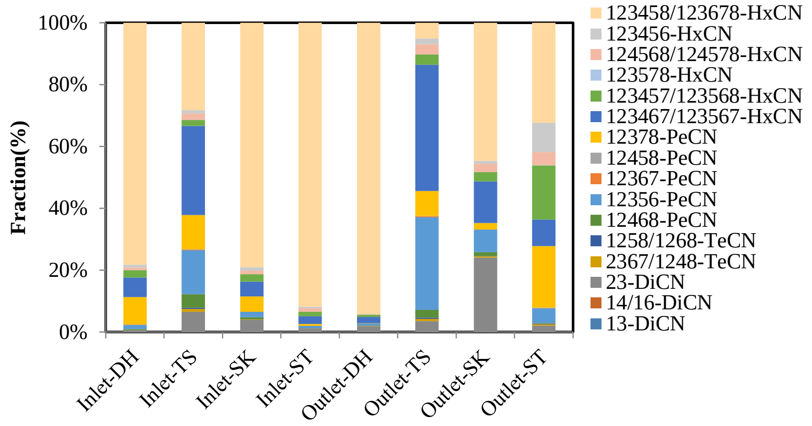

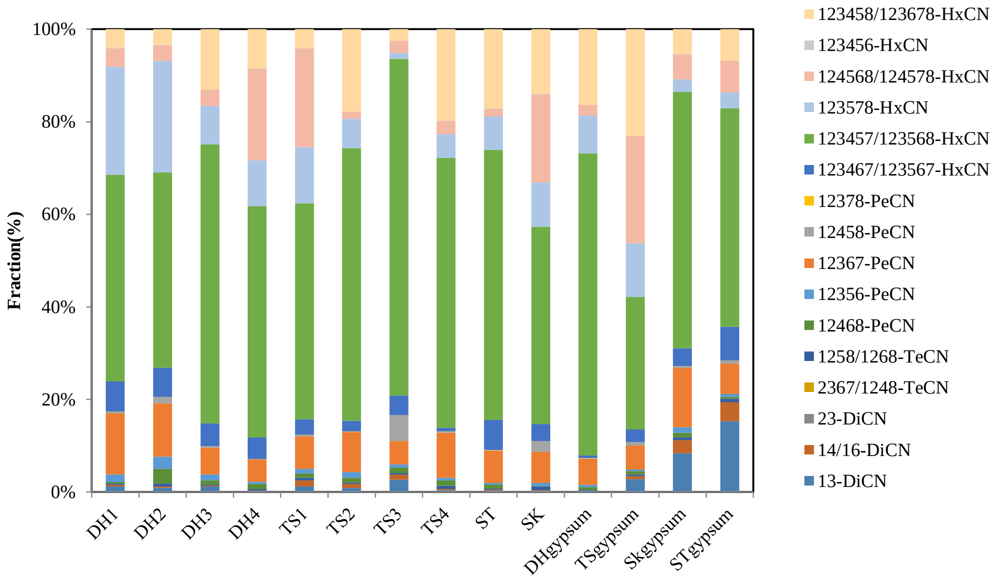

Supplementary Figure S4. TEQ congener patterns of PCNs in the flue gas and gypsum and fly ash samples from sintering plants.

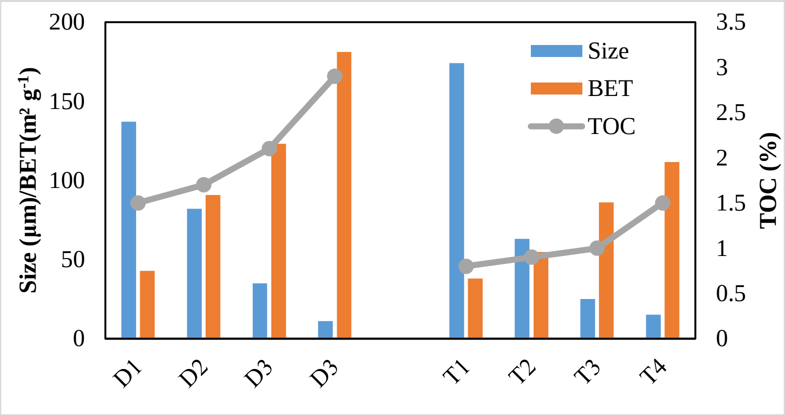

Supplementary Figure S5. Characteristics of fly ash samples from sintering plants. Size=particle size, BET=Brunauer–Emmett–Teller surface area, TOC=total organic carbon.

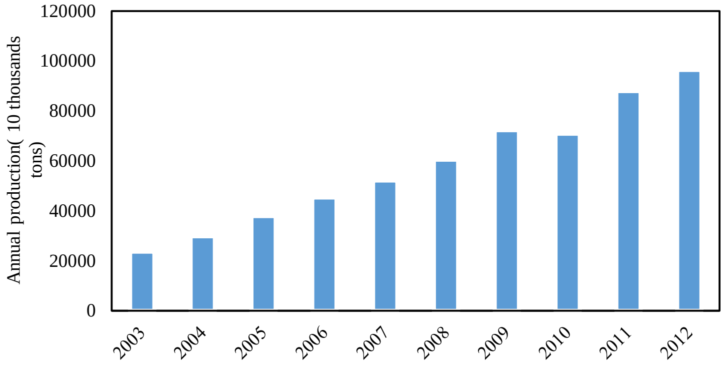

Supplementary Figure S6. The output of sintering in China (2003-2012).
